# Combating Osteoarthritis through Stem Cell Therapies by Rejuvenating Cartilage: A Review

**DOI:** 10.1155/2018/5421019

**Published:** 2018-03-22

**Authors:** Navneet Kumar Dubey, Viraj Krishna Mishra, Rajni Dubey, Shabbir Syed-Abdul, Joseph R. Wang, Peter D. Wang, Win-Ping Deng

**Affiliations:** ^1^Ceramics and Biomaterials Research Group, Advanced Institute of Materials Science, Ton Duc Thang University, Ho Chi Minh City, Vietnam; ^2^Faculty of Applied Sciences, Ton Duc Thang University, Ho Chi Minh City, Vietnam; ^3^Applied Biotech Engineering Centre (ABEC), Department of Biotechnology, Ambala College of Engineering and Applied Research, Ambala, India; ^4^Graduate Institute of Food Science and Technology, National Taiwan University, Taipei, Taiwan; ^5^Graduate Institute of Biomedical Informatics, College of Medical Science and Technology, Taipei Medical University, Taipei, Taiwan; ^6^Department of Periodontics, College of Dental Medicine, Columbia University, New York, NY, USA; ^7^School of Dentistry, College of Oral Medicine, Taipei Medical University, Taipei, Taiwan; ^8^Department of Dentistry, Taipei Medical University Hospital, Taipei, Taiwan; ^9^Graduate Institute of Basic Medicine, Fu Jen Catholic University, Taipei, Taiwan

## Abstract

Knee osteoarthritis (OA) is a chronic degenerative disorder which could be distinguished by erosion of articular cartilage, pain, stiffness, and crepitus. Not only aging-associated alterations but also the metabolic factors such as hyperglycemia, dyslipidemia, and obesity affect articular tissues and may initiate or exacerbate the OA. The poor self-healing ability of articular cartilage due to limited regeneration in chondrocytes further adversely affects the osteoarthritic microenvironment. Traditional and current surgical treatment procedures for OA are limited and incapable to reverse the damage of articular cartilage. To overcome these limitations, cell-based therapies are currently being employed to repair and regenerate the structure and function of articular tissues. These therapies not only depend upon source and type of stem cells but also on environmental conditions, growth factors, and chemical and mechanical stimuli. Recently, the pluripotent and various multipotent mesenchymal stem cells have been employed for OA therapy, due to their differentiation potential towards chondrogenic lineage. Additionally, the stem cells have also been supplemented with growth factors to achieve higher healing response in osteoarthritic cartilage. In this review, we summarized the current status of stem cell therapies in OA pathophysiology and also highlighted the potential areas of further research needed in regenerative medicine.

## 1. Introduction

Osteoarthritis (OA) is a prevalent debilitating joint disorder characterized by erosion of articular cartilage, excessive stiffness pain, and crepitus [1, 2]. According to the United Nations estimates, till 2050, 130 million people will be affected by OA throughout the world, out of which 40 million will develop severe OA [3]. As a consequence, a huge economic pressure will be imposed in treatment and management of OA leading to stressed and decreased quality of life [1, 4]. OA is classified as primary and secondary OA; primary OA is associated with aging, whereas secondary OA is pertinent to disease or other factors [5]. Further, the degradation of network of collagen and proteoglycan in OA cartilage leads to a loss in tensile strength and shear properties of cartilage [6]. Interestingly, though OA manifests as loss of the articular cartilage, it also includes all tissues of the joint, particularly the subchondral bone [5, 7] Besides aging, the increase in level of accumulation of advanced glycation end products (AGEs), oxidative stress, and senescence-related secretory phenotypes are few reported factors associated with pathogenesis of OA [8]. The elevated senescent phenotypes in OA reduces healing properties of cartilage in an aging individual [9, 10], which might be attributed to oxidative damage and telomere shortening [10]. Aging also severely affects extracellular matrix (ECM) and proteoglycans synthesizing capacity of chondrocytes in OA leading to thinning of the cartilage and decreased water content [11–14]. Synthesis of irregular and small aggrecans disrupts the structural integrity of aging cartilage and reduces the chondrocytes' response to cytokines [15].

Currently, the awareness, prevention, diagnosis, and nonpharmacological and pharmacological treatments are used to manage the OA. If these initial nonpharmaceutical interventions fail, the pharmaceutical interventions such as NSAIDs, opioids, and surgery are considered as next level of treatment [16]. However, success of these therapeutic approaches is limited due to related complication and their efficiency. Besides, the autologous chondrocyte implantation (ACI) is one of the most preferred therapeutic approaches for treatment of damaged OA cartilage. Still, the complication related to harvesting chondrocytes had compelled to focus on other cell-based therapies [17]. Recent progresses in tissue engineering have highlighted the regenerative potential of stem cells for therapeutic purposes. The multilineage potential of stem cells, suitable scaffolds, and appropriate chondrogenic agent (chemical and mechanical stimuli) has been implicated to regenerate damaged cartilage [18, 19]. Stem cells could be the unlimited source of chondrocytes and expected to control iatrogenic effects of ACI treatments [18]. Mesenchymal stem cell- (MSC-) based therapy is also emerging as alternative to joint replacement with prostheses, due to its long-lasting effect [20]. The potential of stem cells to differentiate into osteoblasts, chondroblasts, and adipocytes [21], if stimulated properly, can regenerate cartilage both *in vivo* and *in vitro* too [17]. Bone marrow-derived MSC (BMSCs) and the MSCs derived from other cell sources such as synovium, umbilical cord blood, periosteum, peripheral blood, adipose tissue, and muscle have extensively been induced to differentiate into specialized tissues and organs [22]. Moreover, the coculture system of chondrocytes and MSCs have been investigated for cartilage regeneration [17]. Embryonic stem cells (ESCs) are considered as a better source of chondrocytes; however, the ethical concerns and other safety-related complications had impeded the utilization of these cells in regenerative therapy [22]. So, the current researches have more focused towards establishing adult stem cells as therapeutic progenitor for cartilage regeneration. The stem cell-based therapy offers various opportunities such as resurfacing whole joint surface, selection of personalized stem cells, mimicking the environmental conditions to develop the desired phenotype, and increase in level and rate of matrix synthesis, intra-articular stem cell injections, and exogenous wangling of stem cells to regenerate articular cartilage. However, the retention of the chondrogenic phenotype of differentiated stem cells, their integration with native tissue, and mimicking the natural physical strength is posing a challenge to adopt stem cell therapy for OA [18]. Therefore, in this review article, we summarized the current status of stem cell therapies in OA pathophysiology and also discussed the potential areas of further research needed in regenerative medicine.

## 2. Cartilage Injury and Stem Cells

The proper balance of aggrecan and collagen contents establishes the cartilage homeostasis and develops a characteristic physiochemical structure for distribution of loads and mobility [23]. The proteolytic enzymes are associated with synthesis, restructuring and repair of connective tissues, and any cartilage injury or genetic incongruity in association with irregular loading, which promote imbalance in metabolic activity through enhancing proteolytic activity, resulting in degradation of cartilage [24, 25]. Chondrocytes express these proteins under various stimulations such as mechanical stress, oxidative stress, growth factor response, and aging [26]. The cartilage injury leads to disintegration and degradation of cartilage and finally leads to release of aggrecan fragments, chondroitin sulfate, keratin sulfate, and type II collagen along with other catabolic and anabolic products such as disintegrin, the collagenase matrix metalloproteinase 13 (MMP-13), tumor necrosis factor-inducible gene 6 protein (TSG-6), tissue inhibitor of metalloproteinases-1 (TIMP-1), and activin A [27]. Monoclonal antibodies have also been produced to detect the presence of these compounds in body samples such as sera, urine, and synovial fluids of arthritis patients [28–31]. Cartilage is a nonvascular tissue and eludes vascularization by secreting antiangiogenic compounds (thrombospondin-1, chondromodulin-1, and SPARC (secreted protein acidic and rich in cysteine)), collagen type II derived N-terminal propeptide (PIIBNP), and the type XVIII derived endostatin [32]. Along with these factors, presence of tidemarks and calcific nature of the cartilage also resists vascularization of cartilage [33]. It has been established that injured cartilage activates kinases, resulting in activation of growth factors such as fibroblast growth factor 2 (FGF-2) [34, 35] and expression of chemokines and cytokines [27]. FGF-2 plays a critical role in degradation and protection of cartilage depending on its interaction with FGF receptor (FGFR)-1 or FGFR-3, respectively [36]. Molecular signaling pathways such as WNT and BMP have been reported for their role in promoting cartilage repair [37]. It has been reported that cartilage regeneration in OA is promoted by an increase in matrix synthesis and cellular growth, where chondrocytes clumps are generated in middle and deep zones of the cartilage [38, 39]. However, these processes are not capable enough to fully regenerate damaged cartilage [40].

**Figure 1:**
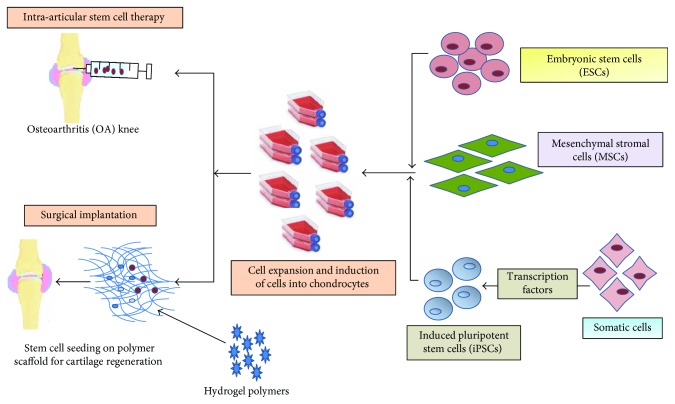
Schematic of stem cell-based therapy in osteoarthritis (OA).

Multilineage potential of stem cells is progressively exploited to regenerate cartilage and to provide cellular therapy for other related arthritis disorders. The current developments in tissue engineering have been made feasible to mimic the process of cartilage synthesis both *in vivo* and *in vitro*. Embryonic stem cells (ESCs), induced pluripotent stem cells (iPSCs), mesenchymal stem cells (MSCs), bone marrow-derived stem cells (BMSCs), adipose-derived stem cells (ADSCs), and synovium-derived stem cells (SMSCs) have been widely explored for regenerating cartilage (Figure 1). In the further section of this article, we will discuss the various stem cells for cartilage regeneration for the treatment of OA.

## 3. MSCs and Cartilage Regeneration

**Figure 2:**
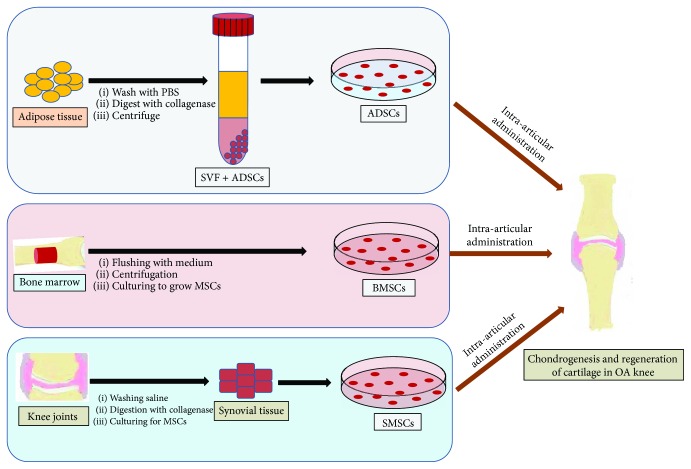
An overview of isolation procedure of various stem cells and their administration in the OA knee joint. OA: osteoarthritis; MSC: mesenchymal stem cells; SVF: stromal vascular fraction; ADSCs: adipose-derived stem cells; BMSCs: bone marrow-derived stem cells.

Owing to multipotency and less problematic with regard to ethical issues, the adult MSCs are the natural choice for cartilage regeneration (Figure 2). The bone marrow, adipose tissues, peripheral blood, umbilical cord blood (UCB), synovium, and skeletal and cardiac muscles are well-known sources of MSCs [41]. Notably, the low concentration of MSCs in bone marrow (BM) aspirate made it compulsory to isolate MSCs which is primarily done by Ficoll gradient centrifugation and further expanded to acquire the sufficient number for quicker recovery of injury after transplantation [42, 43]. The isolated stem cells reduce the chance of cross-contamination and increase the efficacy of stem cell-based therapy. However, the isolation and expansion of MSCs need expertise and also make regenerative therapy expensive. Hence, if the isolation and expansion steps are skipped, it will save significant amount of cost and time in providing cell-based regenerative therapy at less equipped hospitals [44]. To accomplish this goal, volume of BM may be reduced by closed centrifuge to achieve higher concentration of MSCs as compared to Ficoll gradient, which seems a prospective method to provide instant stem cell therapy. Further, the adipose tissue, synovial fluid, and Wharton's jelly of the umbilical cord are considered as potential source of MSCs for cartilage regeneration; however, the source of MSCs depends upon the factors such as feasibility in harvesting, expansion potential, hypoimmunogenicity, and establish procedures [45]. These MSCs are positive for CD73, CD 90, and CD 105 cell markers, whereas they do not express hematopoietic markers such as CD11b, CD14, CD19, CD34, CD45, and HLA-DR [21, 41]. Further, various studies have been carried out to evaluate the potential of human and animal MSCs to regenerate cartilage tissue *in vitro* [46] with reduced immunogenic response [47, 48], thus making feasibility of allogenic MSC transplantation without HLA matching [49]. The other approach for chondrocyte differentiation and cartilage regeneration includes coculturing of MSCs with chondron or other chondrogenesis-promoting cells. Coculturing provides more natural environment and biomechanical stress to promote cartilage regeneration [50, 51]. Various studies have also established an improved chondrogenesis and ECM synthesis when MSCs were cocultured with chondrocytes [52–54].

**Figure 3:**
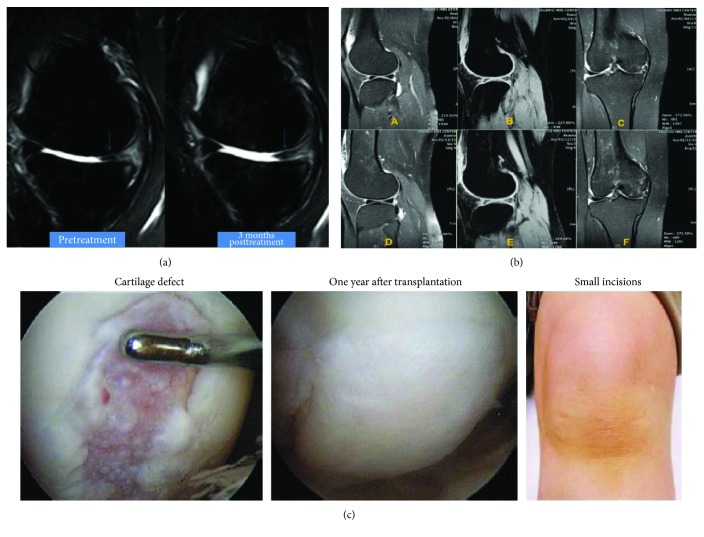
Clinical efficacy of various stem cells-treated OA knee joint: (a) ADSC, (b) BMSC, and (c) SMSC. ADSC: adipose-derived stem cells; BMSC: bone marrow-derived stem cells; SMSC: synovium-derived stem cells; OA: osteoarthritis. Figure 3 is reproduced from Pak [159], Mehrabani et al. [160], and Sekiya et al. [161] [under the Creative Commons Attribution License/public domain].

Cellular contact, secretion of signals (growth factors, cytokines, etc.), and mechanical stress are factors demonstrated to promote cartilage formation and increase in ECM content [55, 56]. Chondrocytes and MSCs in ratio of 1 : 1 and 1 : 4 have been used to explore the advantage of coculture for development of functional cartilage [53, 55, 57]. However, in a study, the coculture of human infrapatellar fat pad-derived stem cells (IPF-ASCs) and chondrons was unable to promote chondrogenic differentiation [58]. BMSCs and articular chondrocytes were cocultured in 1 : 1 ratio in different models and injected in OA-induced rats; as a consequence, the reduced vascularization and hypertrophy along with increased expression of chondrogenic gene was found [59]. Further, in an interesting study, the suspension coculture of hMSCs and hACs was used, which yielded 4.74-fold increase in 3-dimensional aggregates of chondrocytes till 16 days [60]. On the other hand, the hypoxia and transactivation of stable hypoxia-inducible factors (HIF) also promote chondrogenesis [61]. It has also been reported that the proper concentration of hyaluronic acid (HA) also boosts chondrogenesis [62]. Furthermore, the factors including TGF-*β* and insulin growth factor- (IGF-) 1 have been reported to regulate MSC proliferation and chondrocyte differentiation, whereas BMP controls the development of skeletal muscle [63, 64]. Taken together, the MSC-based OA treatment procedures seem promising which has also been shown in various clinical studies (Figure 3). However, some obstacle like control of differentiation, characterization of MSC, and lack of established procedures for chondrogenesis hinders the progress in the therapeutic exploitation of MSCs [65].

## 4. Rejuvenating Cartilage through ADSCs

Stromal vascular fraction (SVF) of adipose tissue contains stem cells, known as adipose-derived stem cells (ADSCs), has the potential to differentiate into chondrocytes, adipocyte, osteoblasts, and myocytes [66, 67]. Currently, the ADSCs are considered as a promising source of chondrocytes due to ease of harvest, their abundance in adipose tissue, and low morbidity rate and side effect, as well as a noninvasive procedure. Various studies have already implicated the significance of ADSC in cartilage regeneration for the treatment of OA [68–71]. Specifically, the intra-articular and surgical implantations of ADSCs combined with biomaterials have been carried out to assess the magnitude of cartilage regeneration in OA-induced animal models [72]. Additionally, the autologous platelet-rich plasma induces cartilage regeneration by secreting growth factors such as TGF-*β*, epidermal growth factor (EGF), and fibroblast growth factor (FGF) to promote the growth and differentiation of stem cells and their adherence to cartilage lesions [73]. In an important study by Tang et al., the intra-articularly injected subcutaneous ADSCs were found to be more effective than visceral ADSC in a rat model of OA [69]. The paracrine effect of ADSCs is considered as one of the paramount factors for cartilage regeneration in OA [74]. Further, the scaffolds seeded with ADSCs in presence of growth factors, stimuli, and compressive stress promote regeneration of cartilage ex vivo. A 3-dimensional scaffold of collagen type I was developed to study the effect of PRP and human recombinant insulin on differentiation of ADSCs into chondrocytes and osteocytes [68]. The study showed that through this approach, it promoted ADSC-mediated chondro- and osteogenesis, and the PRP/insulin-induced differentiation was independent of IGF-1R signaling. In another study, it was reported that xanthan gum significantly improved the chondrogenic potential of intra-articularly implanted ADSC in a rat OA model [70]. Based on the above evidence, ADSC seems highly promising for therapeutic treatment of OA; however, limited knowledge of differentiation mechanism and lack of established procedures are hindering the progress of this therapy to exploit clinically. Issues related to the safety of ADSC-based therapy have been addressed in various clinical trials [75–79]. A report including 70 systematic studies documented that approximately 20% patients developed antibodies during allogeneic cellular therapy, and one case of breast cancer out of 121 patients was also found [75]. Therefore, though the clinical trials indicate the potential of ADSCs in cell-based therapy of OA, further extensive clinical studies are needed to identify potential risks.

## 5. Revitalization of Cartilage by BMSCs

MSCs derived from bone marrow (BMSCs) are capable enough to differentiate into tissues such as bone and cartilage [80, 81] and mobilize at an injured cartilage site in knee joints thereby assisting in cartilage regeneration in OA [82]. In a study, the intra-articularly transplanted BMSC successfully regenerated injured cartilage in a rabbit model of OA and also improved osteoarthritic symptoms in humans without any major side effect even in the long-term [83]. This study demonstrated the possibility of intra-articular injection of MSCs for the treatment of injured articular tissue including anterior cruciate ligament, meniscus, or cartilage. Therefore, if this treatment option is well-established, it may be minimally invasive procedure compared to conventional surgeries. In a very interesting study, out of the alginate, fibrin-alginate (FA), agarose hydrogel 3D culture, and cell pellet systems, the FA hydrogels and cell pellet promoted chondrogenic differentiation of equine BMSCs, whereas no effect was found in agarose group [84]. However, FA seems a better option than pellet culture system, as the pellets require large amount of chondrocytes. Another study established an agarose hydrogel-based model for cartilage regeneration from human BMSCs in presence of TGF-*β*3, where the level of chondrogenesis in agarose gel was dependent on the initial density of cells [85]. Furthermore, a scaffold-free human BMSCs-derived cartilage-like sheet matrix has also been developed in presence of FGF-2 and its efficacy was assessed by transplanting it into an OA rat model. This approach though improved OA condition, the cellular density was decreased significantly within 12 months [86]. Further, in a report by Peng et al., limited proliferation ability of the primary BMSCs was overcomed by immortalizing them by using human papillomavirus- (HPV-) 16 E6/E7 genes, which showed enhanced chondrogenic potential and long-term survival both in *in vitro* and *in vivo* OA mice model [87]. A recent study identified both the promoting as well as the inhibitory role of miR-29b factor in BMSC-based regulation of collagen expression and cartilage regeneration in OA model [88]. Further, the chondrogenically primed BMSCs have also demonstrated to promote cartilage regeneration under hypoxia in a sheep model of OA [89]. However, the effect of oxygen tension was not consistent during ex vivo cartilage regeneration. On the other hand, BMSC also showed enhanced chondrogenesis when seeded on chondrogenic fibrin/hyaluronic hydrogel with improved mechanical strength by adding methacrylic anhydride. Hence, it can also be considered as a promising delivery method for cartilage regeneration in OA therapy [90]. Besides, the intra-articular injection of MSCs may also be applied via microfracture through the cartilage and subchondral bone [91]. In a clinical trial (phase I/II), the intra-articularly injected BMSCs among OA patients showed a significant improvement; however, to assess all the clinical parameters, clinical phase III study was required [92]. In another clinical study, human BMSCs demonstrated that the optimum level of cell dose (25 million) improved the OA without any major adverse effects [93]. However, at higher doses, the knee pain and swelling were among observed as adverse effects, which suggested that more clinical studies are required to establish the therapeutic role of human BMSCs in OA treatment.

## 6. SMSCs and Cartilage Regeneration

SMSCs have been considered more efficient for chondrocyte differentiation than ADSCs or BMSCs [94]. Notwithstanding, in the recent years, only few human-based studies using SMSCs have been conducted compared to ADSCs and BMSC for the treatments for OA. SMSCs are also isolated from hip joints; however, those isolated from knee joints have shown a better chondrogenic potential [95]. These MSCs can also be preserved in complete human serum at 4 or 13°C without significantly affecting their viability and chondrogenic potential [96]. In an interesting research, the exosomes derived from SMSC-140s promoted chondrogenesis without affecting the quality of ECM [97]. SMSCs isolated from OA patients have also shown to be an effective alternative cell source for tissue engineering construct-based therapy for chondral defects [98]. Besides, the pretreatment of SMSCs with IL-1*β* also enhances the chondrogenic potential of SMSCs [99].

In a rat knee OA model, the periodically injected SMSCs migrated into the synovium and retained their undifferentiated SMSC properties with an increased genetic expression of chondroprotective proteins such as BMP-2 and an anti-inflammatory gene, TSG-6 [100]. This suggests that SMSCs not only retain their MSC characteristics but might also inhibit the advancement of OA through genetic machinery. Further, SMSCs have demonstrated the ability to enhance the repair of longitudinally torn menisci in avascular areas in a miniature pig model [59]. In 2014, Hatsushika et al. further demonstrated that intra-articularly injected MSCs in pig knee joint regenerated cartilage in resected medial meniscus [101]. Based on these abovementioned evidence, it could be concluded that the regenerative chondrogenic capabilities of SMSCs catapulted them to the forefront of cell-based OA therapy.

## 7. Infrapatellar Fat Pad- (IFP-) Derived Stem Cells in Cartilage Regeneration

Knee joints are surrounded by extrasynovial adipose tissue known as IFP, which not only provides energy but also releases cytokines/adipokines [102]. IFP is considered as an alternative source of autologous stem cells. MSCs isolated from these tissues of knee joints have superior chondrogenic properties than BMSCs or ADSCs [103]. The characterization of IFP-MSCs is based on the presence of cell markers such as CD9, CD10, CD13, CD29, CD44, CD49e, CD59, CD105, CD106, and CD166 [104]. These cells are also capable to differentiate in trilineages (adipo-, chondro-, and osteogenic) [104–107]. In a recent study, ADSCs were isolated from both the human suprapatellar and IFP and differentiated into trilineage cells. However, the suprapatellar-derived ASCs were found to be more effective in reducing OA symptoms, including knee inflammation and cartilage degeneration in a mouse model [108]. Besides, the IFP-MSCs have been demonstrated with higher rate of expansion compared to synovial fluid- (SF-) MSCs [109]. However, both cells can be exploited to treat the cartilage injury in OA. Further, it was reported that platelet-rich plasma and hyaluronic acid-treated IFP adipocytes promote chondrogenesis and inhibit adipocyte-mediated inflammation [110]. IFP is also a rich source of perivascular stem cells (PSCs) and homeostasis regulating the progenitor MSCs. IFP-PSCs maintain their characteristic and adherent growth properties even after multiple expansion due to their ability to retain the structural integrity of telomere [111]. Interestingly, the PSCs isolated from IFP have shown superior chondrogenic activity as compared to those derived from subcutaneous adipose tissues. In another study, the improved chondrogenic efficiency of coculture of chondrocytes and IFP-MSCs in the presence of chitosan/hyaluronic acid nanoparticles was revealed, which implied that coculture approach in presence of proper stimuli could assist in cartilage regeneration in an osteoarthritic knee [112]. The IFP-PSCs can also be engineered by manipulating the oxygen gradients and mechanical environment of hydrogels to obtain cartilage structurally and functionally similar to a natural one [113]. Though the IFP-derived stem cells appear to be a prospective alternative, further extensive studies are needed to prove their clinical efficacy towards cartilage regeneration for the treatment of OA.

## 8. Regeneration of Cartilage Using ESCs

ESCs are derived from inner cell mass of the blastocyst and could be indefinitely expanded and differentiated into any of the three embryonic germ cell lines including ectoderm, endoderm, and mesoderm [114]. The perpetual self-renewal potential of ESCs makes it unlimited source of stem cells and chondrocytes for cartilage regeneration. However, the major bottleneck to utilize ESCs for cartilage matrix is ethical complexity and poor survival rate of human ESCs after the disintegration of cell mass [115]. Additionally, the differentiation of ESCs into chondrocytes and regeneration of cartilage is complex as it requires complicated microenvironment along with 3-dimensional structure and specific mechanotransduction signal [116]. In a seminal study, McKee et al. showed that under compressive stress, ESCs combined with polydimethylsiloxane (PDMS) scaffolds promoted the initial expression of chondrogenic markers Sox9 and Acan, which further enhanced the expression of collagen type 2 (cartilage-specific marker) and reduced Oct4 (pluripotent marker). However, it did not promote differentiation of hypertrophic cells [116]. This study showed that a proper model is still needed to established ESC-mediated chondrogenesis. An *in vitro* study used embryoid bodies to assess the chondrogenic potential of ESCs and demonstrated that ESCs could develop into hypertrophic and calcifying cells [117]. In another study, ESC also revealed chondrogenic activity when stimulated with bone morphogenetic protein 4 (BMP-4). Further, the accumulation of cartilaginous matrix and type II collagen was recorded in the presence of transforming growth factor- (TGF-) *β*3. [118], and this chondrogenic activity was further promoted by the platelet-derived growth factor- (PDGF-) BB. The higher concentration of BMP-2 with other cofactors, the TGF-*β*-1, insulin, and ascorbic acid, also promotes the chondrogenic ability of ESC under controlled environmental conditions [119]. Transforming growth factor-*β*1_,_ BMP-2, and BMP-4 have been reported to induce differentiation of mice ESCs into chondrocytes [115–120].

Besides, the exosomes have been reported to mediate cellular communication between stem cells and chondrocytes, and understanding this interaction is crucial in developing an effective protocol to regenerate the cartilage [121]. Exosomes are extracellular vesicle primarily secreted by MSCs and assist in maintaining homeostasis, repair and regeneration, and tissue function [122]. In a seminal study, Wang et al. isolated exosomes from culture media of ESC-MSCs and evaluated their effect in OA mice model. This study showed that exosomes exerted protective and regenerative effecst in the injured cartilage [121]. Likewise, various studies have established the chondroregenerative potential of ESCs; however, the major bottlenecks to utilize ESCs for cartilage matrix regeneration are ethical concerns involving the destruction of embryo and poor survival rate of human ESCs after disintegration of cell mass [115, 123].

## 9. iPSCs and Cartilage Regeneration

iPSCs are the reprogrammed somatic cells similar to ESCs, which seems to be a promising alternative to the ESCs [124]. Oct 4, c-Myc, Klf4, Nanog, Esrrb, Lin28, and Sox2 are some of the transcription factors which have been used to reprogram these somatic cells [125–128]. Other approaches like viral transfection and genetic engineering are used to develop iPSCs. Vector characteristics and related promoters are critical factors in gene delivery to differentiate iPSCs into specific cells. Adenoviral, adeno-associated viral, retroviral, and lentiviral vectors have been considered suitable for delivery of target genes in iPSCs [129]. Moreover, the differentiation of patient-specific somatic cell to iPSCs reduces the risk cross-reactivity and immunogenicity [130, 131]. Chondrogenesis has been induced in iPSC-derived embryonic body of mice by using growth factors such as TGF-*β*3, transretinoic acid, and BMP-2 [132, 133]. Another study demonstrated that human iPSCs (hiPSCs) differentiated into chondrocytes and expressed type II collagen and aggrecan similar to cartilage [134]. Zhu et al. induced embryonic body formation from hiPSCs, which were further differentiated to chondrocytes and transplanted in an OA rat to regenerate cartilage [135]. They have also shown that MSCs derived from induced pluripotent cells (iMSCs) were able to secrete exosomes which were superior to exosomes of synovial membrane MSCs (SMMSC) in regenerating cartilage in OA rat [136]. Besides, both with and without scaffold-based cartilage regeneration approaches have been explored for differentiating hiPSCs into chondrocyte for the treatment of cartilage injury [122, 137–142]. The utilization of iPSCs cannot be limited up to regeneration of cartilage but also in the discovery of agents promoting chondrogenesis or inhibiting cartilage degeneration [143]. These studies showed the immense potential of iPSCs to regenerate cartilage in OA. However, the low efficiency and variations in requirement of transcription factors in somatic cells are the major limitation for iPSC generation. Moreover, the undifferentiated iPSC contaminates differentiated MSCs causing tumorigenicity, which limits the use of heterogeneous-differentiated MSCs in cell-based regenerative therapy [144]. Furthermore, over and unregulated expression of *Oct4*, *Sox2*, *Klf4*, and *c–Myc* develops cell dysplasia, serrated polyps and mucinous colon carcinomas, breast tumors, and cancers, respectively [145–150]. Moreover, the clinical application is limited due to lack of a proper model for large-scale and economic differentiation of iPSCs.

## 10. Conclusion and Future Prospects

The self-renewing and multidifferentiation abilities have rendered stem cells, an attractive alternative for the treatment of osteoarthritic pathology. Considering the complexity and efficiency of currently available therapies in long-term, the cell-based regenerative therapy has widely been explored to treat the OA and proven to hold a promising future. The MSCs obtained from adults offer a considerable therapeutic approach in translational medicine. The therapeutic efficacy of stem cells can also be magnified through supplementing growth factors. One of the major limitations of therapies for cartilage repair is that they employ autologous cells and therefore, the development of a universal donor cell is still lacking.

Current reprogrammable approaches to induce stem cell differentiation into cartilage tissues seem inefficient. Further, it seems that genetic modification and gene editing techniques will assist to overcome the current limitations of stem cell-based therapy. The localized delivery of gene therapeutic agents provides more effective and safe recovery in OA. Recombinant adeno-associated viral vector (rAAV) is also used as a genetic vector to deliver genetic sequence in situ to promote the cartilage regeneration [151]. Various animal model studies and clinical trials were carried to out to develop a comprehensive approach for effective gene therapy and encourage to extended clinical trial to develop gene transfer technique to regenerate injured cartilage in situ [152]. microRNAs (miRNAs) such as miR-29a, miR-140-3p, miR-140-5p, miR-145, miR-146a, miR146b, miR-193b, miR-194, miR-221, miR-495 are known to be involved in the differentiation of stem cells into chondrocytes; and their regulated expression enhance chondrogenesis and thus repair cartilage injury [153, 154]. Besides, the development of gene editing technique, the CRISPR/Cas 9 seems promising to regulate chondrogenesis [155]. This technique was exploited in the development of stem cells that controlled interleukin-1 (IL-1) and tumor necrosis factor-*α*- (TNF-*α*-) mediated inflammatory response [156]. Further, the 3-dimensional scaffold promotes the development of cartilage tissue structurally similar to native cartilage by providing conducive microenvironment and essential mechanical stimuli [152, 157]. The recent advances in 3D printing will also improve the scaffold design, which might support chondrocytic growth to overcome osteoarthritic symptoms [158]. It is of note that though multiple studies have sorted out the most effective stem cells, scaffold materials, genetic approach, and other procedures for cartilage regeneration in OA knee and the rigorous randomized and blinded trials, with large sample sizes and long-term follow-up, is needed to reach a consensus.
